# Eine Arachnoidalzyste als Nachahmung einer symptomatischen Alzheimer-Krankheit

**DOI:** 10.1007/s00115-024-01638-0

**Published:** 2024-03-14

**Authors:** Selina Beltrani, Janosch Funk, Johanna Lieb, Michael M. Ehrensperger, Marc Sollberger

**Affiliations:** 1 Memory Clinic, Universitäre Altersmedizin FELIX PLATTER, Basel, Schweiz; 2 Diagnostische und Interventionelle Neuroradiologie, Klinik für Radiologie und Nuklearmedizin, Universitätsspital Basel, Basel, Schweiz; 3 Departement für Neurologie, Universitätsspital Basel, Basel, Schweiz

## Fallbeschreibung

Die 63-jährige Patientin (Rechtshänderin) stellte sich aufgrund seit ca. drei Jahren langsam zunehmenden kognitiven Defiziten ambulant in unserer Sprechstunde vor. Sie berichtete, dass sie (1) ihr unbekannte Wege nicht mehr planen könne, (2) Termine und Abläufe notieren müsse, (3) Probleme bei der Wort- und Namensfindung, (4) dem Lösen komplexer Rechnungen, (5) dem Lesen von Musiknoten und (6) dem Übertrag analoger auf digitale Uhrzeit habe. Zusätzlich bestehe seit ca. einem Jahr eine Unsicherheit beim Treppensteigen.

Drei Jahre vor Beginn der Beschwerden habe sie eine Panikstörung entwickelt. Unter der von ihrer Hausärztin verordneten neuroleptischen Medikation (Deanxit 0,5 mg/10 mg [Flupentixol 0,5 mg/Melitracen 10 mg] 2‑mal täglich) sei sie seit ca. zwei Jahren beschwerdefrei. Weiter nehme sie Ginkgo biloba 240 mg täglich zur Behandlung der kognitiven Beschwerden ein. Die Patientin verneinte Noxen und die Familienanamnese war negativ für demenzielle und psychiatrische Krankheiten sowie für Bewegungsstörungen.

**Abb. 1:**
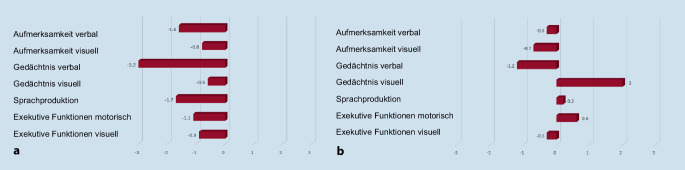
Ergebnisse der kognitiven Tests (anhand z‑standardisierter Werte für Alter, Geschlecht und Ausbildung verglichen zur Normstichprobe der Memory Clinic Basel) **a** präoperativ und **b** postoperativ. Aufmerksamkeit verbal (verbale Merkspanne und Arbeitsgedächtnis aus Wechsler Memory Scale – Revised [WMS-R]); Aufmerksamkeit visuell (visuelle Merkspanne und Arbeitsgedächtnis aus WMS-R); Gedächtnis verbal (Basel Verbal Learning Test: Encodierung, freier verzögerter Abruf, Wiedererkennen); Gedächtnis visuell (Rey Osterrieth Complex Figure: Kopie, freier verzögerter Abruf); Sprachproduktion (verbale Ideenproduktion: Tiere, S‑Wörter; Boston Naming Test); exekutive Funktionen motorisch (Trail Making Test A + B; 5‑Punkte Test); exekutive Funktionen visuell (Stroop Test Durchgang 1, Durchgang 3/1)

Es erfolgte eine neuropsychologische Untersuchung mit Prüfung der verbalen und visuellen Aufmerksamkeit, des verbalen und visuellen Gedächtnisses, der Sprachproduktion sowie der verbalen und motorischen Exekutivfunktionen [2]. Im Vergleich zur Normstichprobe der Memory Clinic Basel [3] fanden sich schwere Defizite im verbalen Gedächtnis (Encodierung, freier verzögerter Abruf), bei ansonsten leichten Minderleistungen in der verbalen Aufmerksamkeit (Merkspanne, Arbeitsgedächtnis) und der Sprachproduktion (verbale Ideenproduktion; Abb. 1a).

Der von einer Fachärztin für Neurologie supervidierte neurologische Status blieb unauffällig, insbesondere fehlten Zeichen einer Stand- und/oder Gangstörung. Psychopathologisch zeigte sich eine depressive Stimmungslage (Beck-Depressions-Inventar [6] Wert mit 7/63 Punkten unauffällig; einzelne Haupt- und Zusatzkriterien einer depressiven Episode nach ICD-10 bejaht, ohne jedoch die Kriterien einer depressiven Episode gesamthaft zu erfüllen). Die serologischen Abklärungen (Hämogramm, Elektrolyte, Leber- und Nierenwerte, Schilddrüsen-stimulierendes Hormon (TSH), Folsäure, Vitamin B12, Vitamin D) waren unauffällig.

Zusammengefasst war das klinische Bild, unter Berücksichtigung der anamnestischen Angaben, des klinischen Verlaufs und der im Vordergrund stehenden verbalen Abrufstörung suggestiv für das Vorliegen einer Alzheimer-Krankheit im Frühstadium.

**Abb. 2:**
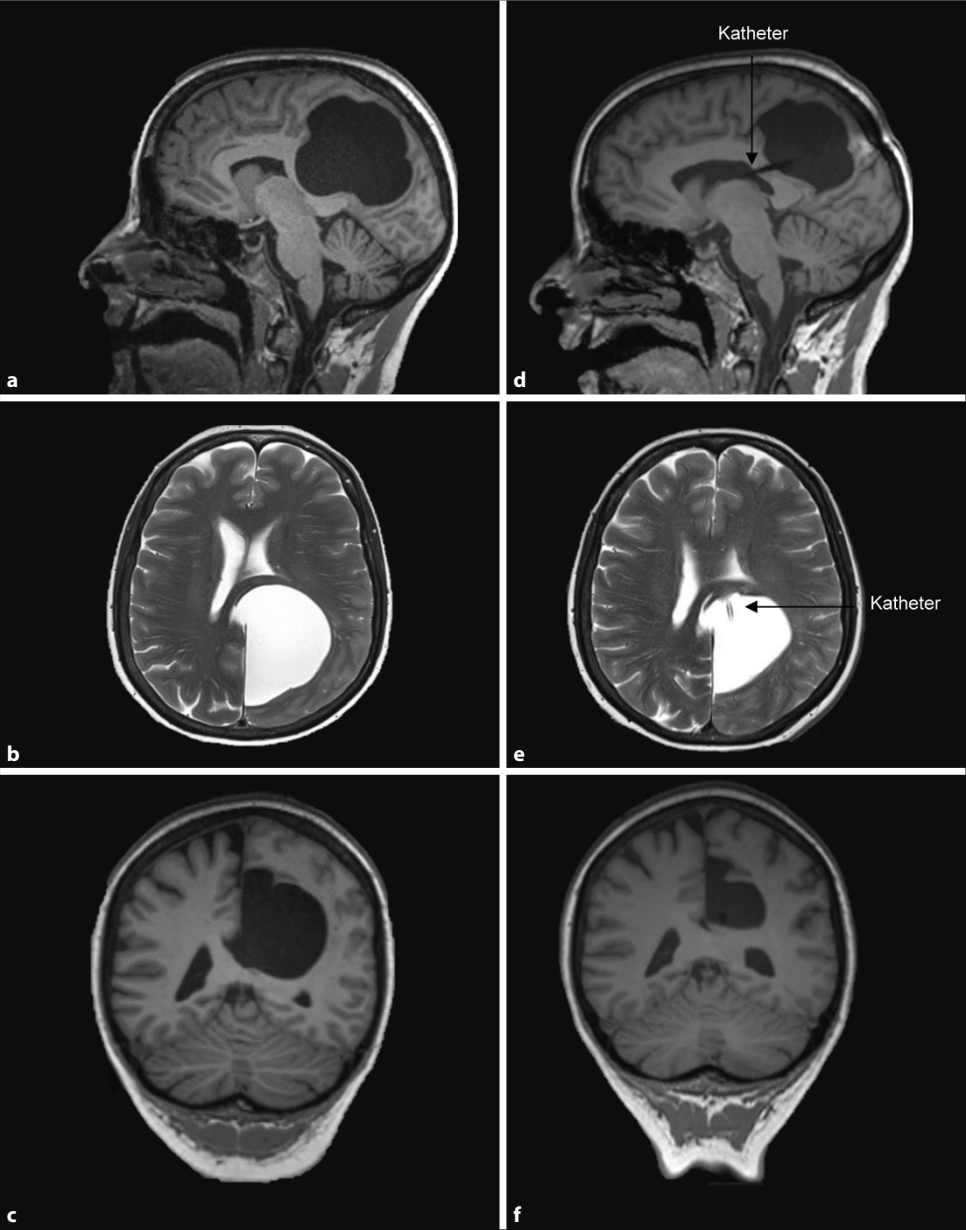
Magnetresonanztomographie des Neurokraniums (**a,** **d** T1w sagittal; **b,** **e** T2w axial; **c,** **f** T1w koronar) präoperativ (**a–c**) und drei Monate postoperativ (**d–f**)

Gemäß der S3-Leitlinie Demenzen von 2016 führten wir als nächstes eine Magnetresonanztomographie (MRT) des Neurokraniums durch. Diese zeigte eine Arachnoidalzyste (max. 6,2 cm, Volumen von 99 cm^3^) links mit raumforderndem Effekt auf den Parietallappen, inklusive dort verstrichener Gyri und Sulci, Pelottierung des linken Seitenventrikels, des Splenium corporis callosi sowie leichter Distension des linken Temporalhorns (Abb. 2a–c). Dagegen fand sich visuell und volumetrisch (VEOmorph, VeoBrain GmbH ©) keine fokale oder globale Hirnatrophie.

Bei mit dem kognitiven Ausfallsprofil zu vereinbarender Lokalisation der raumfordernden Arachnoidalzyste und hohem Leidensdruck der Patientin erfolgte die Entlastung mittels Zystenfenestrierung und Einlage eines zystoventrikulären Katheters (Abb. 2d–f).

Zwei Wochen nach der operativen Entlastung berichtete die Patientin von einer beinahe vollständigen Rückbildung ihrer kognitiven Beschwerden und ihrer Unsicherheit beim Treppensteigen. Die MRT-Verlaufskontrolle drei Monate postoperativ zeigte eine deutlich größenregrediente Arachnoidalzyste (max. 5,4 cm, Volumen von 51 cm^3^) mit einer Volumenreduktion von ca. 50 % und rückläufig raumforderndem Effekt auf den Parietallappen (Abb. 2d–f). Die neuropsychologische Verlaufskontrolle erfolgte sechs Monate postoperativ und ergab, passend zu den anamnestischen Angaben und der Verlaufsbildgebung, eine beinahe vollständige Rückbildung der kognitiven Defizite (Abb. 1b). Eine MRT-Verlaufskontrolle knapp eineinhalb Jahre postoperativ zeigte eine weitere Regredienz des Zystenvolumens auf nun 40 cm^3^.

## Diskussion

Arachnoidalzysten sind seltene (0,3–1,7 % [1]), meist angeborene, gutartige, mit Liquor gefüllte intrakranielle Hohlräume [11]. Sie sind typischerweise asymptomatisch. In einer Fallserie mit 661 Erwachsenen mit Arachnoidalzysten wurden in einzig 5 % der Fälle Symptome berichtet [1]. Das Spektrum der Symptome ist sehr breit und reicht von neurologischen Störungen wie Kopfschmerzen, Gangunsicherheit, Inkontinenz oder epileptischen Anfällen über affektive Störungen wie Psychosen bis hin zu kognitiven Störungen wie Wortfindungsstörungen, Verlangsamung oder Gedächtnisdefiziten [1, 11].

Gemäß einer Fallserie mit 299 Erwachsenen treten Arachnoidalzysten mehrheitlich (66 %) temporal und nur selten (12 %) wie in unserem Fall parietal auf [7]. In der Literatur fanden wir zwei Fallberichte, welche kognitive Defizite bei einer symptomatischen Arachnoidalzyste links parietal beschrieben [8, 9]. Bei beiden Patientinnen lagen ähnliche Symptome wie bei unserer Patientin vor; darunter langsam fortschreitende Namens- und Wortfindungsstörungen. Unsere Patientin berichtete zusätzlich über visuell-räumliche Schwierigkeiten, wie der Planung einer Wegstrecke, sowie über Unsicherheit beim Treppensteigen. In den genannten Fallberichten erfolgte keine neuropsychologische Untersuchung, was ein Vergleich der kognitiven Störungsbilder verunmöglicht.

Interessanterweise zeigte sich bei unserer Patientin als Ausdruck des Schweregrades der Raumforderung der linksparietalen Zyste, eine bihemisphärische, wenn auch deutlich linksbetonte kognitive Funktionsstörung mit im Vordergrund stehenden verbalen Gedächtnisdefiziten im Sinne einer Abrufstörung. Das visuelle Gedächtnis war präoperativ zwar normgerecht, zeigte sich postoperativ aber vergleichbar verbessert wie das verbale Gedächtnis.

Der (posteriore) parietale Kortex wird traditionell in Verbindung mit Aufmerksamkeitsprozessen, der Perzeption und der sensomotorischen Verarbeitung gesehen [10]. Gestützt auf funktionielle MRT-Studien gibt es jedoch Hinweise auf eine Aktivierung dieser Struktur beim freien Abruf von Gedächtnisinhalten (siehe z. B. [4]). Gemäß des Attention to Memory Model kann demnach eine Abrufstörung als Folge einer Störung des Zusammenspiels von Top-down (konzeptgesteuert, Gedächtnissuche nach bestimmten Vorgaben) und Bottom-up (reizgesteuert, Erkennung von verhaltensrelevanten Reizen in der Umgebung) Aufmerksamkeitsprozessen interpretiert werden [4]. Im Gegensatz dazu zeigt sich beim Vorliegen einer Alzheimer-Krankheit, mit meist primärer Funktionseinschränkung im medialen Temporallappen, typischerweise neben einer Abrufstörung auch eine fehlende Speicherung der Gedächtnisinhalte [10].

Die von der Patientin wahrgenommene Unsicherheit beim Treppensteigen bildete sich nach erfolgter operativer Entlastung zurück, was für einen Zusammenhang dieses Symptoms mit der Arachnoidalzyste spricht. Es ist nicht ungewöhnlich, dass Arachnoidalzysten zu Störungen führen, welche sich teils unspezifisch präsentieren und sich, wie unserem Fall, neuroanatomisch nicht klar zuordnen lassen [11].

Ein Zusammenhang zwischen der vorbekannten Panikstörung und der Arachnoidalzyste scheint unwahrscheinlich. Neben der fehlenden zeitlichen Assoziation zwischen Panikstörung und kognitiver Störung fehlt auch der neurofunktionelle Zusammenhang. Beim Vorliegen einer Panikstörung ist von einer Funktionsstörung des Angstnetzwerkes mit Fokus in der Amygdala auszugehen, nicht jedoch von einer Affektion parietaler Strukturen [5].

## Fazit für die Praxis

Unser Fall zeigt die Reversibilität einer organisch bedingten kognitiven Störung, was die Wichtigkeit der frühzeitigen Diagnostik kognitiver Beschwerden unterstreicht. Daneben zeigt er beispielhaft die Bedeutung sorgfältiger neurologisch-psychiatrischer und neuropsychologischer Untersuchungen unter Einbezug neuroanatomischen Wissens zum gezielten Einsatz apparativer Diagnostik und letztendlich therapeutischer Maßnahmen.
